# Tumors of the Central Nervous System: An Update

**DOI:** 10.3390/cancers12092507

**Published:** 2020-09-03

**Authors:** Carla Mucignat-Caretta

**Affiliations:** Department of Molecular Medicine, University of Padova, Via Marzolo, 3-35131 Padova, Italy; carla.mucignat@unipd.it

The brain may be affected by a variety of tumors of different grade, which originate from different cell types at distinct locations, thus impacting on the brain structure and function. In 2016, the classification of brain tumors by the World Health Organization underwent a reappraisal, focused on improving differential diagnosis through the combined use of histological, molecular and genetic indicators [1].

The *Cancers* Special Issue “Tumors of the Central Nervous System: An Update” lists 21 contributions, including 14 research papers and 7 reviews, from different areas of research, from bench to bedside. These contributions are intended to give a timely overview of the most recent development on cell mechanisms and on possible interactions among different cell types or brain structures, in order to improve our strategies to combat these diseases. This may be accomplished through better understanding the growth of the different tumors, the impact on brain and body functions, and lastly the development of clinical issues, from improving diagnosis and prognosis to chemo- and radiotherapy. 

For its fast pace, aggressive development and dismal prognosis, the best known and most studied tumor of the brain is glioblastoma. While other malignant brain tumors, different from glioblastoma, may be as devastating, the effect of benign tumors in the brain may also be harmful, due to interactions with areas controlling vital functions. Hence, cell and tissue research may improve our approach to tumors to hopefully eliminate them or at least block their growth, preserving patient’s health, safety and well-being. 

In this Special Issue, several topics are discussed and new data are presented. A general principle in cancer development is outlined in the review by Roelecke and colleagues [2], which describe how tumor cells communicate with the microenvironment through dynamic membrane structures named tunneling nanotubes and tumor microtubes, events that may lead to resistance to radio- and chemotherapy. These structures may represent a new avenue to cancer treatment. 

An overview of different therapeutic options for glioblastoma treatment is presented by Rajaratnam and colleagues [3], together with a glimpse on the pathogenesis of this tumor. This includes isocitrate dehydrogenase (IDH) mutations, and the role of various signaling pathways like Notch, ceramide, vascular endothelial, epidermal, and platelet-derived growth factors, PI3K/AKT/mTOR, PTEN and SHH. However, other genes may participate in determining the outcome of glioblastoma: by examining a panel of 409 genes, it was shown that hypermutated yet IDH-wild type patients younger than 55 years had a better prognosis [4]. The impressive growth rate of glioblastoma is best understood if we refer not only to the single tumor cell behavior, but to the effect of glioblastoma cells on the upregulation of autophagy in pericytes of neighboring non-tumor areas, which decrease immune function and promote tumor growth [5].

A crucial issue in the development of ground-breaking therapies is the availability of innovative models to closely mimic the disease. In this context, the development of I. genetically engineered mice using Cre/LoX for selective cell targeting, II. improved transposon technology for transgene integration and III. CRISPR-Cas9 knockouts paves the way to improved testing of new therapeutic targets [6]. In addition to genetic engineering technologies, mice can also be employed to host patients-derived xenografts, to provide a clinically relevant model that was used to explore the efficacy of inhibitors of cyclin-dependent kinase, proteins that are deeply involved in driving the development of glioblastoma [7]. 

Interestingly, there are different genetic signatures that may increase the risk of glioblastoma development: a meta-analysis on more than 15,000 cases reveals how three types of associations are linked to an increased susceptibility to the development of all types of glioma, or to gliomas associated to mutant or wild-type IDH [8]: this classification may steer both diagnosis and therapeutic perspectives. Furthermore, the different aggressiveness of gliomas may be linked to specific molecular features, in detail, it has been connected to the EGFR-TMEM167A-p53 pathway. The increased aggressiveness of wild-type p53 gliomas is possibly related to enhanced growth factor signaling through the effect of TMEM167A in the modulation of vesicular trafficking [9]. One of the most reliable traits among the different molecular phenotypes linked to glioblastoma severity is the methylation status of the O6-methylguanine DNA methyltransferase (MGMT) promoter, which is linked to the improved response of glioblastoma to temozolomide therapy. Notably, data analysis shows that methylation status may change between primary tumor and relapse [10], with implications for treatment and prognosis. The same feature, MGMT promoter methylation, appears as a strong prognostic factor also for another brain glioma, namely pilocytic astrocytoma, since it reduces the chance of recurrence, so that this index may have prognostic validity in addition to the location of the tumor and the possibility of complete resection [11].

An improved prognostic definition is strongly desirable for patients and prompts for the quest of new and reliable molecular cues. In this framework, recent developments point to the interactions between different cells that may result in the release of molecules or vesicles, that may alert on the presence of one type of tumor. These can be easily detected in fluids like the cerebrospinal fluid, thus overcoming, at least in part, the necessity for biopsies. Exosomes actually mediate the interactions between glioma stem cells and the surrounding environment by increasing tumor aggressiveness. In this process, the role of Semaphorin 7A on exosome surface is to interact with integrin Beta-1 to promote cell migration [12]. Taking advantage of an already known solid tumor marker, named metastasis-associated in colon cancer-1 (MACC1), the accuracy of survival prediction has been improved by using the plasma level of these transcripts in addition to IDH mutation status, with wildtype IDH1 and high MACC1 delineating the worst scenario [13]. Another very interesting possibility is to identify the circulating microRNA, so that miRNA in the cerebrospinal fluid have been used in a monocentric study to accurately differentiate healthy subjects and different types of brain cancer, including glioblastoma, low-grade glioma, meningioma and brain metastases according to their miRNA portfolio [14]. This strategy could help in better defining uncertain diagnoses posed through imaging, reducing the necessity for biopsies. Furthermore, the serum levels of circulating DNA, both cell-free and from exosomes, reporting the V600E mutation in the BRAF gene, have been deployed as strong indicators for central nervous system tumors in children [15]. This possibility is appealing for brain tumors, in particular for follow-up in children or for monitoring response to treatments, since repeated exposure to biopsy procedure may not be desirable. However, it is noteworthy to recall that the disease development and progression is not only a matter of tumor cells and microenvironment, but has to do also with the general health status of the patient, in particular in aging persons: to better define prognosis in elderly glioblastoma patients, the Comprehensive Geriatric Assessment has been validated as an independent predictor of survival in fit, vulnerable and frail patients [16].

To defeat glioblastoma, an approach combining resection, chemotherapy and radiotherapy is usually devised. However, the identification of new therapeutic targets is highly desirable. The overexpression of P-glycoprotein may reduce the accumulation of anticancer drugs within the tumor cells, including glioblastoma. Novel pyrazolo[3,4-d]pyrimidines compounds acting on this protein result in increased accumulation and enhanced efficacy of anticancer drugs, while maintaining favorable pharmacokinetics and tolerability [17]. Another molecular target for glioblastoma treatment has been selected through gene expression analysis that identified the RNA-binding ubiquitin ligase MEX3A as strongly expressed in glioblastoma: it acts by binding and ubiquitinylating the tumor suppressor RIG-I thus addressing it to degradation [18]. Moving to cancer-promoting phenomena, inflammation is on the spot. Anakinra, an antagonist to interleukin-1 receptor in use for autoinflammatory diseases, is able to decrease proinflammatory genes expression also in glioblastoma, reducing also migration and proliferation [19].

In addition to the development of new drugs, a better understanding of radiotherapy efficacy is also seminal for a successful treatment. This is particularly true for glioblastoma, since radioresistance related to the presence of tumor stem cells hampers the complete efficacy of radiotherapy. Long-term effects of hypofractionated treatment on glioma stem cells are apparent on proliferation and in gene expression profile, affecting the modulatory pathways of apoptosis and differentiation [20].

Lastly, cutting-edge technologies are fundamental for an accurate diagnosis at first occurrence, but also to predict recurrence. The use of diffusion tensor imaging and fractional anisotropy maps is particularly challenging in edematous areas which surround glioblastoma tissue: by using a deep learning paradigm for correcting free water signals, it was possible to correctly predict glioblastoma recurrence using preoperative data from 35 patients [21]. Actually, various deep-learning strategies are available to reduce uncertainty related to edema, angiogenesis and necrosis, which are challenging in glioblastoma imaging. By combining pre- and post-surgery imaging with genetic characterization and follow-up information, it is now possible to predict survival in both preclinical and clinical settings [22].

In conclusion, while research is continuously moving forward, we offer this issue as a step towards improvement of patient health, starting from molecular and technological advancements that hopefully will jump into the clinic, through the open discussion of controversial topics and the recognition of actual advancements.
